# Ubiquitylation of Rad51d Mediated by E3 Ligase Rnf138 Promotes the Homologous Recombination Repair Pathway

**DOI:** 10.1371/journal.pone.0155476

**Published:** 2016-05-19

**Authors:** Deqiang Han, Junbo Liang, Yalan Lu, Longchang Xu, Shiying Miao, Lin-Yu Lu, Wei Song, Linfang Wang

**Affiliations:** 1 Department of Biochemistry and Molecular Biology, State Key Laboratory of Medical Molecular Biology, Institute of Basic Medical Sciences Chinese Academy of Medical Sciences, Peking Union Medical College, Beijing, China; 2 Department of Cancer Genetics and Epigenetics; Beckman Research Institute; City of Hope, Duarte, California, United States of America; 3 Key Laboratory of Reproductive Genetics, Ministry of Education and Women's Reproductive Health Laboratory of Zhejiang Province, Women's Hospital, School of Medicine, Zhejiang University, Hangzhou, Zhejiang, China; 4 Institute of Translational Medicine, Zhejiang University, Hangzhou, Zhejiang, China; The University of Hong Kong, HONG KONG

## Abstract

Ubiquitylation has an important role as a signal transducer that regulates protein function, subcellular localization, or stability during the DNA damage response. In this study, we show that Ring domain E3 ubiquitin ligases RNF138 is recruited to DNA damage site quickly. And the recruitment is mediated through its Zinc finger domains. We further confirm that RNF138 is phosphorylated by ATM at Ser124. However, the phosphorylation was dispensable for recruitment to the DNA damage site. Our findings also indicate that RAD51 assembly at DSB sites following irradiation is dramatically affected in RNF138-deficient cells. Hence, RNF138 is likely involved in regulating homologous recombination repair pathway. Consistently, efficiency of homologous recombination decreased observably in RNF138-depleted cells. In addition, RNF138-deficient cell is hypersensitive to DNA damage insults, such as IR and MMS. And the comet assay confirmed that RNF138 directly participated in DNA damage repair. Moreover, we find that RAD51D directly interacted with RNF138. And the recruitment of RAD51D to DNA damage site is delayed and unstable in RNF138-depleted cells. Taken together, these results suggest that RNF138 promotes the homologous recombination repair pathway.

## Introduction

Cells have developed intricate DNA damage response to sense and repair DNA-damaging lesions induced by endogenous and exogenous insults. Life-threatening DNA double-strand breaks (DSBs) can be repaired primarily by non-homologous end joining (NHEJ) and homologous recombination (HR) [1–3]. In response to DSBs, a series of proteins of cell cycle checkpoint signaling and DNA damage repair are recruited hierarchically to lesion sites. Reversible protein post-translational modifications (PTMs) tightly regulates these recruitments [4].

Ubiquitylation is a dynamic and reversible PTM that regulates a wide range of biological processes, including cell cycle progression, transcription, apoptosis and inflammation [5]. Increasing evidence demonstrates that ubiquitylation is a critically important component in orchestrating DNA damage signaling and repair processes through regulating protein stability, localization and activity [5–7]. Central components to this process are RNF8 and RNF168 ubiquitin ligases. The E3 ubiquitin ligase RNF8 binds to phosphorylated MDC1 at DSB sites as a deck to trigger the recruitment of another E3 ubiquitin ligase RNF168 [5]. Ubiquitylation of H2A mediated by RNF168 provides an additional recruitment signal for RNF168, allowing sustained recruitment of repair factors, such as BRCA1 and 53BP1. In addition, RNF169 has been found to participate in regulating the early ubiquitylation signaling cascade initiated by RNF8 and RNF168 [8, 9]. RFWD3 is another RING ubiquitin E3 that has been shown to promote p53 stability following DNA damage [10–12].

The choice between NHEJ and HR pathways is made through processing the broken DNA ends. HR requires forming a single-stranded DNA (ssDNA) overhang at the break, a process known as resection. In mammalian cells, resection is sensed and mediated by the MRE11-RAD50-NBS1 complex (MRN), CtIP, BLM and EXO1[13–15]. RAD51 recombinase is essential to the HR reaction and assembles into helical polymers wrapping around the ssDNA tail at the lesion site [16]. Next, heterotrimetic replication protein A (RPA) is accumulated to the ssDNA ends which are aligned to the RAD51 helical nucleoprotein filament to form an intact homologous DNA molecule. Assembly of RAD51 monomers to ssDNA is facilitated by several mediator proteins. BRCA2 is an important loader of RAD51 monomers at lesion sites. In addition, RAD51 recruitment also depends on RAD52 and RAD51 paralogs which is a family of five proteins including XRCC2, XRCC3, RAD51B, RAD51C and RAD51D [17, 18].

Our previous study found E3 ligases RNF138 is mainly expressed in testis and might be involved in regulating early stages of development. RNF138 is a 245 aa ubiquitin E3 ligase with a RING domain, zinc fingers, and an ubiquitin interacting motif (UIM), which was originally found to act as a negative regulator of Wnt signaling through interacting with NLK. Here we identified RNF138 as a DNA damage response protein that is recruited to DNA damage site very quickly through its Zinc finger domains. We found that RNF138 was a substrate of ATM and could be phosphorylated at Ser124. Depletion of RNF138 dramatically affected the RAD51 assembly at DSB sites after irradiation (IR). This suggested that RNF138 was involved in regulating HR. Furthermore we found RNF138 directly interacted with RAD51D using modified tandem affinity purification technology.

## Materials and Methods

### Plasmid, constructs and siRNA

Human RNF138 full length gene, RING domain, Zinc finger domain deletion mutants and human RAD51D gene were cloned into pEGFP-N1, pCMV-HA pCMV-myc or p 3xFlag CMV14 vectors. Point mutants of RING domain and Zinc finger domains were constructed using site directed mutation procedure with pEGFP-N1/RNF138. All the plasmids obtained were sequenced to confirm that there were no mutations in the coding sequences. The siRNA sequences targeting RNF138 is GGAUCACUGUAACAGUAAUTTAUUACUGUUACAGUGAUCCTT. siRNAs were transfected into cells using oligofectamine (Invitrogen) according to manufacturer’s instructions.

### Cell culture, antibody, immunoprecipitation and western blotting

Human cell lines U2OS, HEK293T and HCT116 were purchased from ATCC. MDCI-, H2AX-, RNF8-, 53BP1- deficient MEFs and wild-type MEF were gereruously supplied by Dr. Xiaochun Yu described previously[19]. Human cell lines and MEF cells were maintained in DMEM medium with 10% fetal calf serum and cultivated at 37°C in 5% CO2 (v/v). Cells were lysed with NETN buffer (contains 0.5% NP40,50 mM Tris-HCl pH 8.0, 100 mM NaCl, 2 mM EDTA) with Roche Protease Inhibitor Cocktail. Immunoprecipitation and western blotting were performed following standard protocol as described previously. Rabbit anti-RNF138 antibody is purchased from Santa Cruse, anti-glutathione s-transferase (GST) antibody, monoclonal anti-β-actin antibodies and GAPDH antibody were purchased from Sigma. Mouse monoclonal anti-GFP antibody and Phospho-(Ser/Thr) ATM/ATR Substrate antibody were purchased from Cell Signaling Technology. RPA antibody was purchased from Abcam. γH2AX, RAD51, RNF8, MDC1, BRCA1, Rabbit anti-53BP1 antibody was kind gifts from Dr. Xiaochun Yu.

### Stable cell line establishment, complex purification and Mass Spectrometric Analysis

For the establishment of HEK293 cell lines of stably expressing epitope strep-flag tagged RNF138 with silent mutation of shRNA target sequence combined with shRNA sequence under additional U6 promoter, RNF138 sequence was first silently mutated the RNF138 shRNA target sequence from G GAT CAC TGT AAC AGT AAT to A GAC CAT TGC AAT AGC AAC. Next, the mutant sequence fused with strep tag and flag tag was cloned into modified pIl3.7 vector as well as RNF138 target shRNA sequence under additional U6 promoter. Briefly, 40μg of the expression plasmid and packaging vector were transfected into HET293 cells in 15 cm plates. Culture supernatants were then filtered using 0.45 μm filters after 48 h virus secretion. HEK293 cells were infected with the viral supernatant was diluted four fold in fresh medium. Next, cells were dissociated into a single-cell suspension using trypsin. FACS was performed using a BD Aria II sorterto sort the positive cells that expressed GFP.

The stable cell lines were lysed in lysis buffer (50 mM Tris-HCl (pH 7.4), 100 mM NaCl, 0.5%NP-40, 1 mM EDTA and protease inhibitor cocktails) and subjected to affinity purification described previously. The purified protein complex was resolved on SDS-PAGE and Coomassie stained with Coomassie R250. The distinct protein bands were excised manually. And the samples were pretreated, and mass spectrometry analysis was performed as described previously[20].

### Laser microirradiation and microscope image acquisition

Cells transfected with corresponding plasmids were grown on 35-mm glass bottom dishes (Corporation). Laser microirradiation was performed on OLYMPUS IX71 inverted fluorescence microscope coupled with the MicroPoint Laser Illumination and Ablation System (Photonic Instruments, Inc.). A 337.1 nm laser diode (3.4 mW) transmits through a specific Dye Cell and then yields 365 nm wavelength laser beam that is focused through X60 UPlanSApo / 1.35 oil objective to yield a spot size of 0.5–1 μm The pulse energy is 170 μJ at 10 Hz. Images were taken by the same microscope with CellSens software (Olympus). The GPF fluorescence strips were recorded at indicated time points and then analyzed with ImageJ software. 20 cells were analyzed from three independent experiments. Error bars represent the standard deviation.

### Immunofluorescence staining

For immunofluorescence staining, cells were treated with laser microirradiation or different doses of irradiation and washed 3 times by PBS. Next, the cells were fixed in 3% paraformaldehyde for 5 min and permeabilized with 0.5% Triton X-100 in phosphate-buffered saline (PBS) for 5 min at room temperature. Samples were blocked with 8% goat serum and then incubated with the primary antibody for 1 h. Samples were washed for three times and incubated with the secondary antibody for 45 min. After three times of washing by PBS, 100 μl DAPI was added. Next, the coverslips were mounted onto glass slides and visualized with OLYMPUS IX71 inverted fluorescence microscope.

### Colony formation assay

U2OS transfected with control siRNA or RNF138 siRNA were plated in the wells of a 6-well plate, each 1000 cells, immediately after MMS and radiation. After incubation for 10 days, the surviving cell fractions were calculated by comparing the numbers of colonies formed in the cultures plates. For rescue assay, the colony formation assay was performed with U2OS transfected with RNF138siRNA 48 hours and with wild type RNF138 plasmid.

### Chromatin fraction

Cells were harvested following 10 Gy of IR treatment and washed twice with PBS. Subsequently cell pellets were suspended in the NETN buffer (20 mM Tris-HCl, pH 8.0, 100 mM NaCl, 1 mM EDTA and 0.5% NP-40) and incubated on ice for 15 min. Insoluble fraction was recovered and suspended in 0.2M HCl. The soluble fraction was neutralized with 1 M Tris–HCl pH 8.0 for further study.

### Comet assay

U2OS were transfected with control siRNA or RNF138 siRNA. After 24 h, Cells were irradiated with or without 5 Gy of IR and recovered in normal culture medium for indicated time at 37°C. Cells were collected and washed twice with ice-cold PBS; 10^4^/ml cells were combined with 1% Low melting point agarose at 40°C at the ratio of 1:3 (v/v) and immediately pipetted onto slides. The slides were immersed in the alkali lysis solution (1.2 M NaCl, 100 mM EDTA, 0.1% SDS and 0.26 M NaOH) 3 h at 4°C Then, the slides were subjected to electrophoresis at 15 V for 25 min (0.6 V/cm) and stained in 10 mg/ml Propidium iodide for 20 min. All images were taken with a fluorescence microscope and analysed by Open Comet plugins of ImageJ software.

### Gene convention assay

U2OS cells stably expressing the integrated homologous recombination reporter DR-GFP were transfected with RNF138 siRNAs or control siRNA for 48 h, and infected with or without I-SceI-expressing adenovirus. Cells were harvested after 24 h, then fixed and quantitated by flow cytometry.

## Results

### Rnf138 accumulates to laser-induced DNA damage sites rapidly

**Fig 1:**
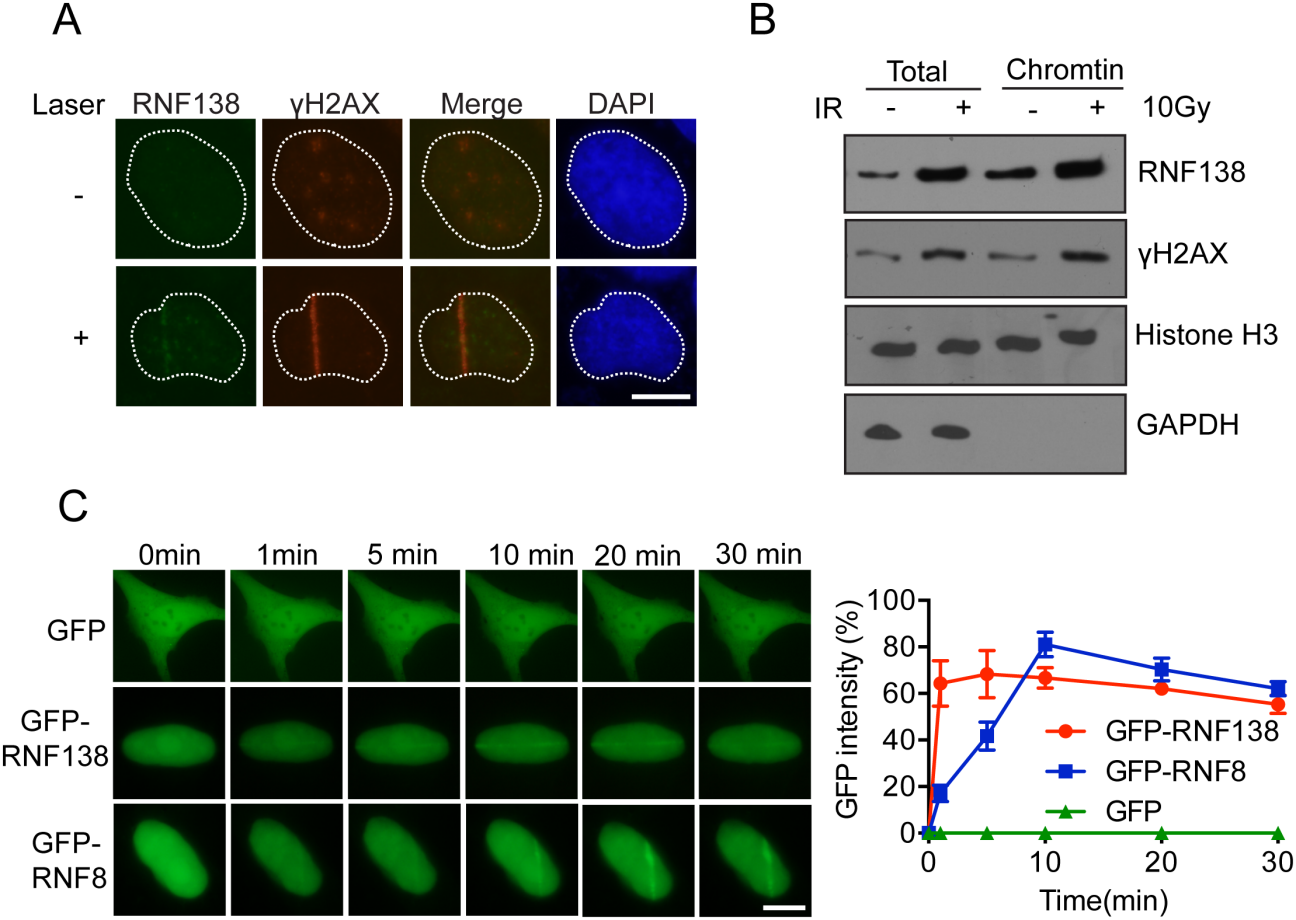
RNF138 accumulates to laser-induced DNA damage site rapidly. (A) Flag tagged RNF138 (Flag-RNF138) co-localized with γH2AX to laser irradiated DNA damage tracts. U2OS were transfected Flag-RNF138 and treated with laser microirradiation. Immunofluorescence staining was performed use Flag and γH2AX antibody. (B) Endogenous RNF138 accumulated on chromatin following DNA damage by IR. (C) The relocation kinetics of GFP-RNF138 and GFP-RNF8 recruited to DNA damage sites. GFP tagged RNF138 and RNF8 were expressed in U2OS, and the relocation kinetics was monitored in a time course following laser microirradiation. GFP is taken as negative control. GFP signal intensities at the laser line were converted into a numerical value using ImageJ software. Normalized fluorescent curves from 20 cells were averaged. The error bars represent the standard deviation. Signal intensities were plotted using Graphpad Prism software.

DNA damage related proteins often exhibit dynamic mobilization following DNA lesion. To investigate whether RNF138 participates in DNA damage response, we first determined whether it could accumulate at DNA damage sites. Interestingly, Flag-tagged RNF138 became concentrated at lesion site overlapped with the recruitment of DNA damage marker γH2AX following laser microirradiation treatment (Fig 1A). Next, we assessed the subcellular distribution of RNF138. It was further confirmed by detecting endogenous RNF138 of chromatin fraction that RNF138 was increasing following induction by ionizing radiation (Fig 1B). In untreated cells, GFP-tagged RNF138 proteins were mostly localized in the nucleus (Fig 1C). Laser microirradiation can generate localized trace of DNA damage in live cells in which protein kinetics can be monitored by fluorescence microscopy. So we transfected GFP-RNF138 into U2OS cells and monitored the localization kinetics of RNF138 in response to laser-induced DNA damage. Interestingly, time-lapse imaging showed that GFP-RNF138 was efficiently recruited to sites of laser-induced DNA damage (Fig 1C). According to the time course, RNF138 recruited to DNA damage site lasted as quite a long period as GFP-RNF8. RNF8 is the major ubiquitin E3 ligase responsible for ubiquitylation during DNA damage response. However, RNF138 recruitment to DNA damage was not dependent on RNF8, which suggests that RNF8 and RNF138 participate in different DNA damage pathways.

### Rnf138 can be phosphorylated by ATM at Ser124

**Fig 2:**
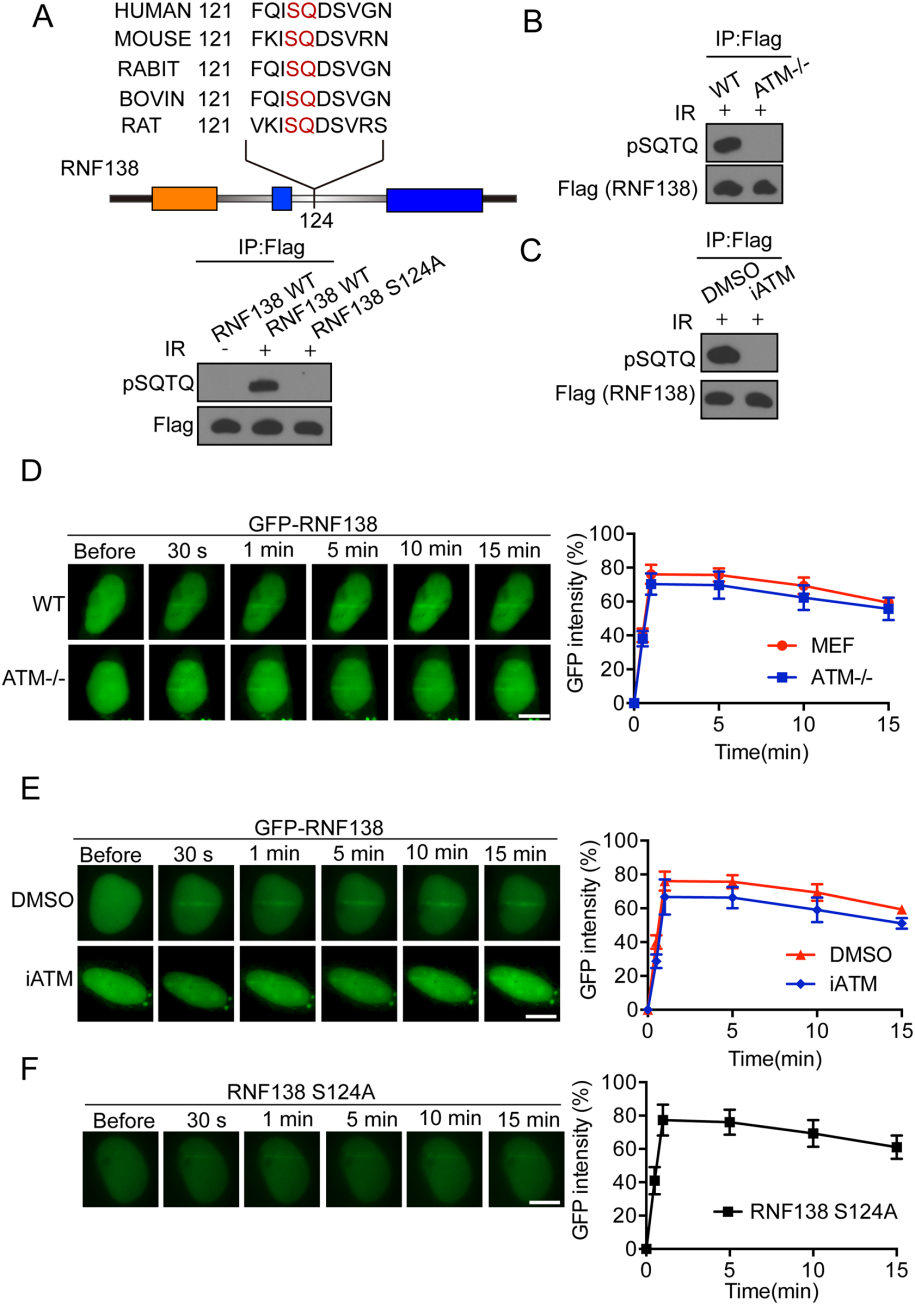
RNF138 can be phosphorylated by ATM at Ser124. (A) Confirmation of ATM-dependent phosphorylation of RNF138 at Ser124 sites after exposure of cells to IR. Flagged RNF138 wild-type and Point mutation at S124A transfected in HEK293T cells treated with or without IR. Sample were performed IP and analyzed by Phospho-(Ser/Thr) ATM/ATR Substrate Antibody. (B) Phosphorylation of RNF138 abolished in ATM deficient cell. ATM deficient MEF cells or wild-type MEF cell transfected Flagged RNF138 were exposed to IR (5 Gy). Cells were lysed and performed IP and analyzed by Western blot with phospho-(Ser/Thr) ATM/ATR substrate antibody. (C) Phosphorylation of RNF138 abolished by ATM inhibitor. Same as (B), except that cells were pre-incubated with DMSO or 10 μM KU55933 for 1 h before exposure to IR. (D) Recruitment of GFP-RNF138 to DNA damage sites in ATM deficient MEF cells. GFP-RNF138 was expressed in ATM deficient MEF or wild-type MEF, The relocation was monitored in a time course following laser microirradiation. (E) The effect of ATM inhibitor KU55933 treatment on the recruitment of GFP-RNF138 to DNA damage sites. GFP-RNF138 was expressed in U2OS and treated with 10 μM KU55933 for 1 h, The relocation was monitored in a time course following laser microirradiation. (F) The relocation kinetics of GFP-RNF138 S124A mutant recruited to DNA damage sites. The relocation was monitored in a time course following laser microirradiation. Scale bar = 10 μm. For quantitative and comparative imaging, signal intensities at the laser line were converted into a numerical value using ImageJ software.

We found that RNF138 could be recruited to laser microirradiation-induced DNA damage site. Some protein kinases such as ATM and ATR mediate the cellular DNA damage response signaling through phosphorylation of substrate. We analyzed the amino acid sequence of RNF138 and found that RNF138 contained one conservative potential SQ site at Ser124 (Fig 2A). Since RNF138 might be a candidate substrate of ATM [21], we first tested if RNF138 could be phosphorylated under DNA damage. 293T cell were transfected with flag-tagged RNF138 following 1 h exposure to 10Gy IR radiation, and then the phosphorylation status of RNF138 with immunoprecipitation by M2 flag antibody was examined by immunostaining with Phospho-(Ser/Thr) ATM/ATR Substrate Antibody(anti-pSQ/TQ). Phosphorylation of RNF138 was observed following IR radiation (Fig 2A). Further, we mutated supposed phosphorylation site of RNF138 residues Ser-124 to Alanine using site-directed mutagenesis. As expected, Ser-124 mutation totally abolished phosphorylation of RNF138 (Fig 2A). Provided that the central role of ATM in DNA damage relates to phosphorylation, we next tried to confirm whether the phosphorylation of RNF138 was processed by ATM. We treated the 293T cells transfected flag-tagged RNF138 with ATM kinase inhibitor (KU55933). ATM kinase inhibitor totally abolished phosphorylation of RNF138 (Fig 2B). Additionally, there was no phosphorylation of RNF138 detected in ATM knockout cell (Fig 2C). We further examined whether recruitment of RNF138 to DNA damage site requires its phosphorylation by ATM by monitoring the time course of RNF138 during laser microirradiation. GFP-tagged RNF138 was transfected to U2OS cells, which were then treated with ATM kinase inhibitor for 1 hour. Imaging results showed that GFP-RNF138 were recruited to laser-induced DNA lesion sites. And the efficiency of recruitment to lesion sites was constant and the same as that observed from the untreated cells (Fig 2D). Consistently, ATM knockout cells transfected with GFP-tagged saw RNF138 concentrated on DNA lesion sites (Fig 2E). Next, we found that RNF138 S124A mutant was still recruited to DNA damage sites (Fig 2F). These findings indicate that RNF138 is a substrate of ATM, and phosphorylation of RNF138 by ATM is dispensable for its recruitment to the DNA damage site.

### Accumulation of Rnf138 at DNA damage site through its zinc finger domains

**Fig 3:**
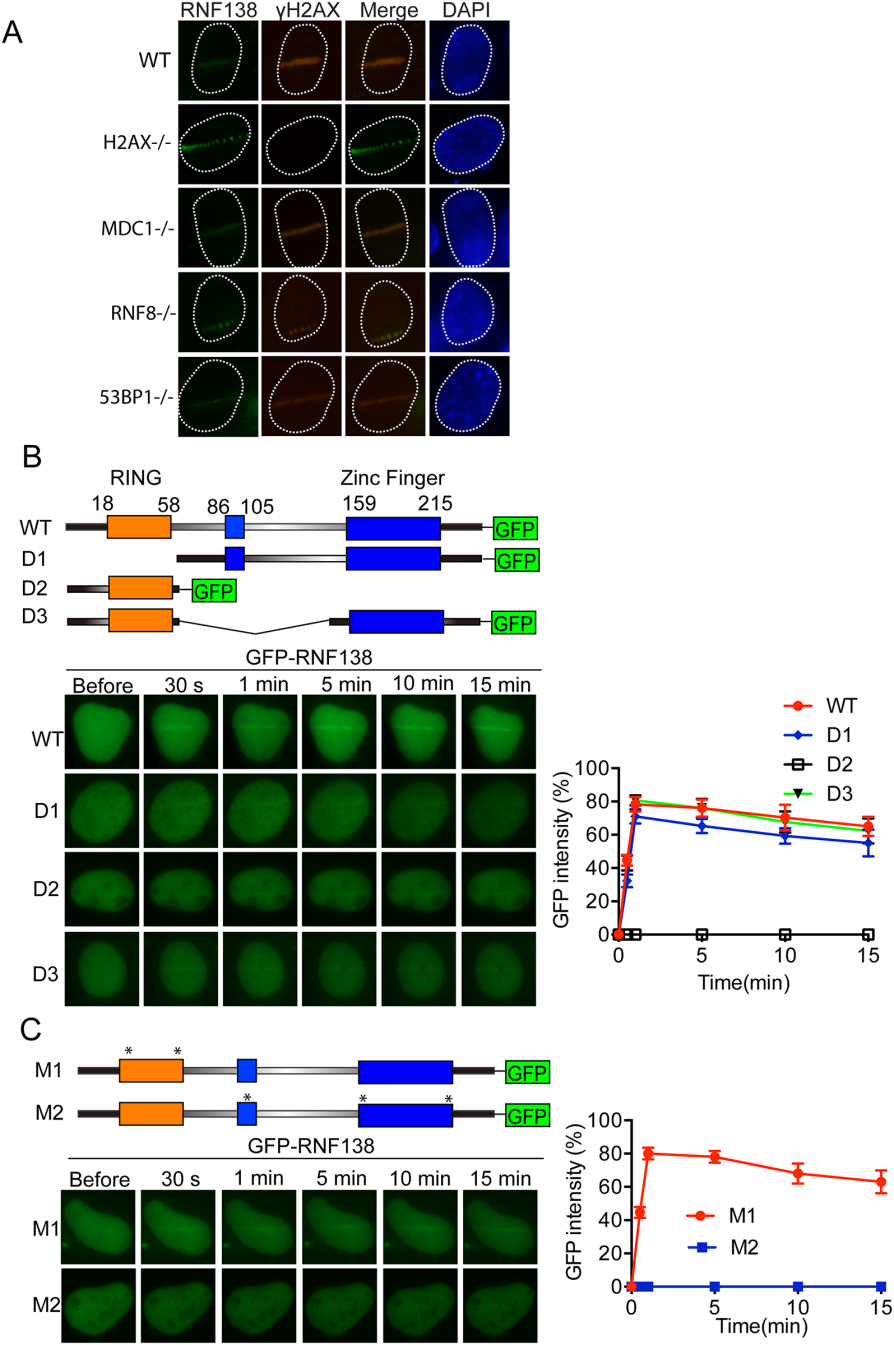
Accumulation of RNF138 at DNA damage site through its Zinc Finger domains. (A) RNF138 co-localize with γH2AX to laser irradiated DNA damage in DNA damage response related gene knockout MEF cells. γH2AX-/-, MDC1-/-, RNF8-/-, 53BP1-/- gene knockout MEF cells was transfected Flag-RNF138 and treated with laser microirradiation. Immunofluorescence staining was performed use Flag and γH2AX antibody as shown. (B) Upper panel: Schematic diagram of protein domain structure of RNF138 and deletion mutation. Lower panel: the relocation kinetics of RNF138 and indicated deletion mutants to sites of laser-induced DNA damage after micro-irradiation in U2OS cells. (C) Upper panel: Schematic diagram of point mutation of protein domain of RNF138. M1 is mutated with C18A, C54A; M2 is mutated with C86A, C159A and C189A. Lower panel: The relocation kinetics of RNF138 and indicated point mutants to sites of laser-induced DNA damage after micro-irradiation in U2OS cells. Scale bar = 10 μm. For quantitative and comparative imaging, signal intensities at the laser line were converted into a numerical value using ImageJ software.

To delineate where RNF138 participates in the established DNA-damage signaling cascade, we examined Laser microirradiation induced-recruitment of RNF138 in a number of cells with deficiencies in various DNA-damage checkpoint proteins and sensor proteins. Our anti-RNF138 antibody could not detect endogenous RNF138 in mouse embryonic fibroblasts (MEFs), so we transfected flag-tagged RNF138 into MEF (Fig 3A). Formation of Laser-induced RNF138 recruitment was not noticeably affected in MDCI-, H2AX-, RNF8-, BRCA1-, 53BP1-deficient MEFs comparing with the wild type MEF.

We next sought to confirm what affected the recruitment. RNF138 was characterized as E3 ligase with an amino-terminal RING finger domain and three C2HC or C2H2 zinc fingers domain (Fig 3B). We generated a series of truncation and internal deletion mutants of RNF138 with GFP tag in C terminal. We transfected these mutants into U2OS cells and monitored the kinetics of their recruitment, respectively. Compared with kinetics of wild type, the RING domain deletion mutant (D1) only slightly delayed recruiting to the DNA lesion sites. Interestingly, the mutant without zinc fingers and only with the RING domain (D2) could not recruit to DNA damage sites. Mutants with single deletions in C2HC zinc finger domain (D3) did not affect RNF138 recruitment (Fig 3B). To further confirm the results, we mutated RING domain or zinc fingers domains respectively, kinetics of recruitment of RNF138 was consistent with its deletion mutants (Fig 3C). These results indicate that zinc finger domains of RNF138 are important for its recruitment to DNA damage site.

### Rnf138 is involved in regulating HR repair pathway

**Fig 4:**
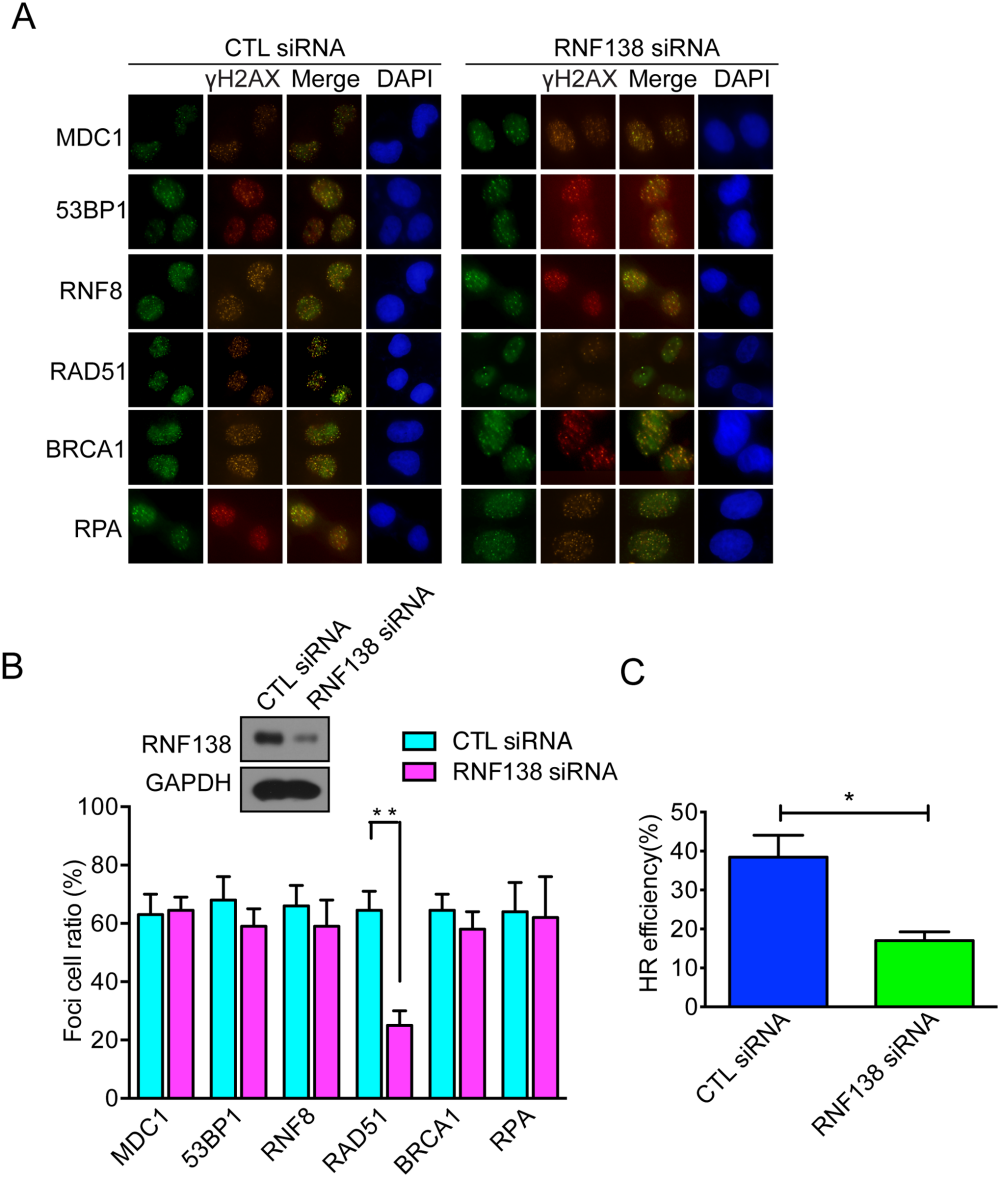
RNF138 involve in regulating HR repair pathway. (A) Foci formation of γH2AX, MDC1, 53BP1, RNF8, RAD51, BRCA1 and RPA in RNF138 deficient cells or control cells. Hela cells transfected with RNF138 siRNA exposed to 8Gy IR, Immunofluorescence staining was performed use γH2AX, MDC1, 53BP1, RNF8, RAD51, BRCA1 and RPA antibody as shown. (B) Relative quantification of the blots of Foci formation.The percentage of cells with Foci plotted and statistical analysed using GraphPad Prism 5. 50 cells were analysed for each experiment. Scale bar = 10 μm. Error bars represent the SD, n = 3. (C) HR efficiency was measured by HR-mediated gene conversion assay. DR-GFP U2OS cells were transfected with RNF138 siRNA and infected with adenovirus expressing I-SceI. Approximately 10,000 cells were evaluated by flow cytometry in each experiment. Error bars represent the SD n = 3.

To further depict what role of RNF138 in the DNA-damage signaling pathway, we analyzed whether depletion of RNF138 affected foci formation of different DNA-damage checkpoint proteins and sensor proteins. Indeed, we found that RNF138 depletion significantly decreased RAD51 foci formation, whereas no significant impact was observed on foci formation of,MDC1, 53BP1, RNF8, RAD51, BRCA1, and RPA (Fig 4A and 4B). Due to the importance of RAD51 in homologous recombination of DNA during double strand break repair, the RNF138-depedent decrease of RAD51 assembly at DSB sites suggested that RNF138 should take part in regulating HR repair. To confirm RNF138 's participation in regulating HR repair, we performed I-SceI mediated gene conversion assay to measure HR efficiency in DR-GFP cells with or without depletion of RNF138. Interestingly, efficiency of HR decreased in RNF138-depleted cells by approximately 50% (Fig 4C). Taking these results together, we concluded that RNF138 is involved in regulating HR repair pathway.

### Rnf138 participates in DNA damage repair

**Fig 5:**
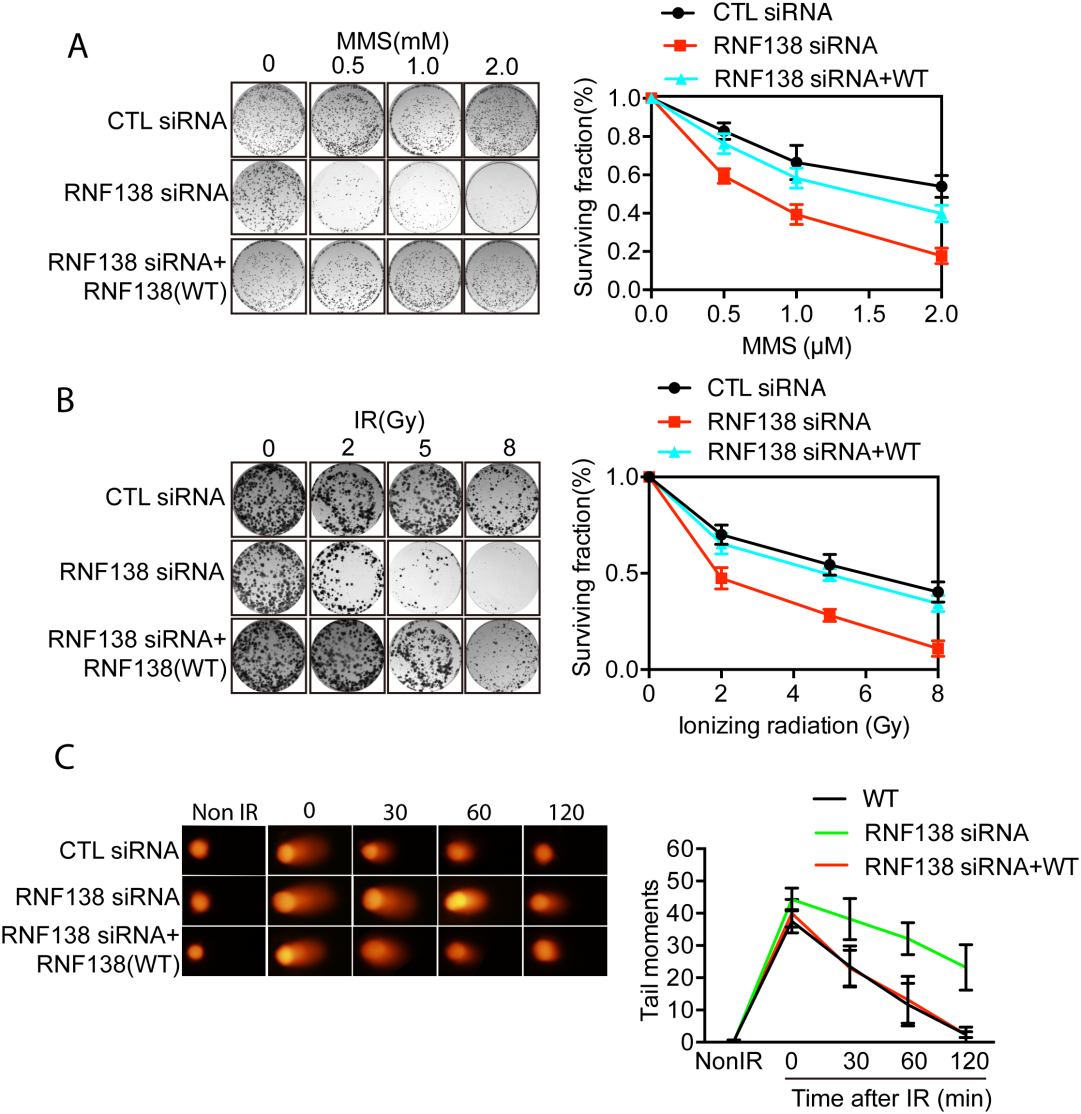
RNF138 participates in DNA damage repair. (A) The viability of RNF138-depleted cells to different dose of IR. Hela cells were treated with control or RNF138 siRNAs then exposed to various doses of IR. Error bars represent the SD. n = 3. (B) The viability of RNF138-depleted cells to different dose of MMS. Hela cells were treated with control or RNF138 siRNAs and treated with various doses of MMS. Error bars represent the SD. n = 3. (C) Cells with indicated genotypes were subjected to comet assays at indicated time points following 5 Gy of IR treatment. Tail moments were summarized from three independent experiments with at least 50 cells in single time point per sample. Error bars represent the SD. n = 3.

To further confirm the role of RNF138 in the DNA damage response, we treated RNF138 depleted cells and control cells with difference doses of MMS or ionizing radiation (IR) and then analyzed their viability. Interestingly, RNF138 depleted cells were hypersensitive to IR or MMS in dose-dependent manner (Fig 5A and 5B). In addition, the sensitive phenotype of RNF138-depleted cells could be rescued by transfecting wild type RNF138 constructs. We next measured DNA damage repair kinetics by comet assay under alkaline condition. As shown in Fig 5C, within 2 h following low dose IR treatment (5Gy), most DNA damage sites were repaired in wild-type Hela cells, but not in RNF138-depleted cells. Moreover, RNF138-depleted cells were reconstituted with wild- type RNF138. These results suggested that RNF138 participates in DNA damage response.

### Rnf138 ubiquitylates Rad51d to facilitate HR repair pathway

**Fig 6:**
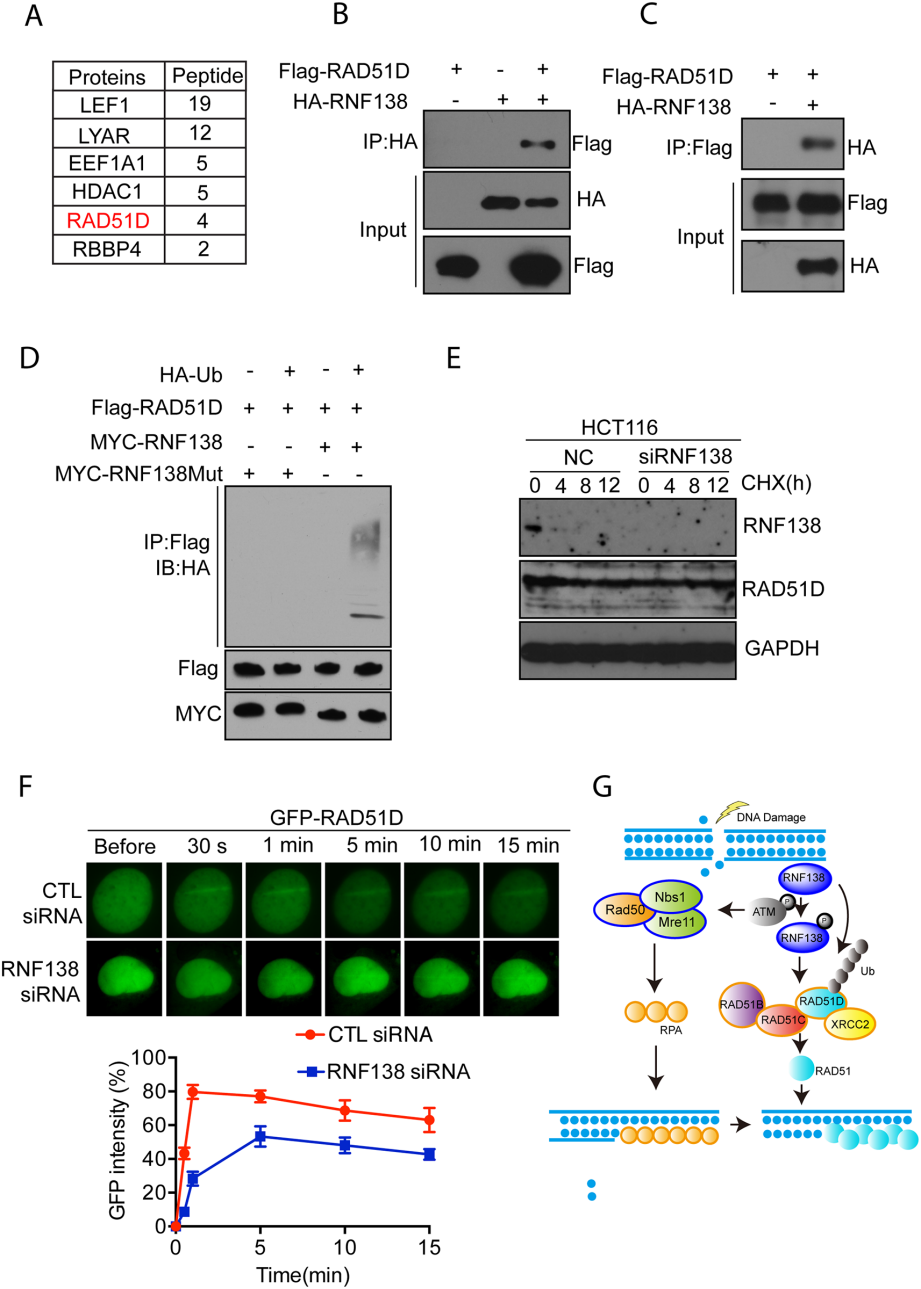
RNF138 ubiquitylates RAD51D to facilitate HR repair pathway. (A) Analysis of RNF138 protein binding partners using modified tandem affinity purification. The data from mass spectrometry analysis are presented in the tables. (B)(C) Interaction between RNF138 and RAD51D was confirmed by Co-immunoprecipitation. (B) and Reciprocal co-immunoprecipitation (C) of RNF138 and RAD51D from 293T cell extracts. (D) Poly-ubiquitylation of RAD51D mediated by RNF138 dependent on its RING finger E3 ligase activity. (E) Endogenous RAD51D degradation was chased at indicated time points after treaded with Cyclohexamide (CHX) in RNF138 deficient HCT116 cells and control cells. (F) The relocation kinetics of GFP-RAD51D in RNF138-depleted cells and control cells. GFP tagged RAD51D was expressed in RNF138-depleted U2OS or control cells, and the relocation kinetics was monitored in a time course following laser microirradiation. (G) Schematic overview of RNF138 regulating homologous recombination repair pathway.

In an attempt to identify potential targets of RNF138 that might regulate the HR repair pathway and further elucidate the mechanism of this process, we performed modified tandem affinity purification using stably expressing epitope strep-flag tagged RNF138 with silent-mutated shRNA target sequence connecting in series with an shRNA sequence under an additional U6 promoter in 293 cells. One homologous recombination related protein, RAD51D, was identified by Mass spectrometry (Fig 6A). Co-immunoprecipitation of HA-RNF138 and myc-RAD51D performed in HEK293T cells further confirmed the interaction between RNF138 and RAD51D (Fig 6B). Reciprocal interaction between RNF138 and RAD51D was detected (Fig 6C). These results suggested that RNF138 and RAD51D interact with each other. As RNF138 is an E3 ligase, next we performed ubiquitylation assay in the presence of recombinant HA-ubiquitin. As shown in Fig 6D, RAD51D was ubiquitylated by RNF138, which depended on the E3 ligase activity at its RING domain. However, we knocked down the RNF138 using siRNA in 293T to analyze protein dynamics of RAD51D, there was no obvious change in half-life of Rad51D observed compared with negative control (Fig 6E). These results showed that ubiquitylation of RAD51D mediated by RNF138 should be a regulatory signal rather than a proteolytic degradation pathway. To test this we transfected GFP-tagged RAD51D into U2OS with or without RNF138 targeting siRNA. The relocation kinetics of GFP-RAD51D was monitored in a time course following laser microirradiation. Interestingly RAD51D was dramatically delayed to the lesion sites in RNF138-deficent cells (Fig 6F). RAD51D and related paralogs (RAD51B, RAD51C, RAD51D and XRCC2) are required for homologous recombination DNA repair; hence, shortening the retention of RAD51D at laser-induced DNA damage sites implied that RNF138 is required for the RAD51D, facillitating RAD51 faliment formation at DNA lesion sites.

## Discussion

DNA damage-dependent ubiquitylation has an important role as a signal transducer in the DNA DSB response. In this study, we highlight that RNF138 is one of the E3 ligases recruited to DNA damage sites which is mediated by its zinc fingers. Our results demonstrate that RNF138 is involved in regulating homologous recombination repair. We modified the tandem affinity purification technique combined with Mass spectrometry. This model stably expresses epitope strep-flag tagged RNF138 with a silent-mutated shRNA target sequence with a target RNF138 shRNA. By using this approach, RNF138 related proteins could be enriched.

Our Multiple experiments confirmed that RNF138 directly interacts with RAD51D. RAD51D is one of the five-protein family called RAD51 paralogs. These proteins mainly function through forming complexes, not just self-assembly of individual paralogs [22]. These proteins were identified through sequence-similarity searches of damage-response genes. They share approximately 20–30% identity at amino acid level with RAD51 and with each other. Most of the RAD51-like proteins have been shown to have DNA-stimulated ATPase activity and preferentially bind to single-stranded (ss)DNA [23, 24]. Many studies have supported that RAD51 paralogs are required for homologous recombination DNA repair. RAD51D and p53 deficient mouse cells have an increased frequency of chromosome aberrations [25]. According an animal model study, RAD51D deficient mice died between 8.5 and 11.5 dpc, and the Embryos displayed a range of abnormalities [26]. RAD51 paralog deficiency abrogates RAD51 induced foci formation in response to IR [27], suggesting a role for these proteins in facilitating RAD51 assembly at sites of DSBs. Rad51D deficient cells were previously reported to be most sensitive to DNA damage insults [18]. Interestingly, efficiency of homologous recombination decreased observably in RNF138-depleted cells. And RNF138-depleted cells was hypersensitive to DNA damage insults, such as IR and MMS. These findings strongly support the connection between RNF138 and RAD51D and call for further elucidation of the molecular mechanisms and function of RNF138 in its relation to HR DNA damage repair.

In addition, RAD51D can be ubiquitilated by RNF138 *in vivo*. When E3 ligase linked ubiquitin chains were added to a substrate, protein degraded through the proteasomal complex or interacted with each other in a proteasome-independent mannter. The non-degradative ubiquitylation is particularly important in regulating protein activity, stability and localization. This study found that ubiquitylation of RAD51D mediated by RNF138 acts in a non-degradative way in DNA damage response. RNF8 and RNF168 have been shown to promote non-degradative ubiquitylation of histones and subsequent recruitment of DSB repair factors such as BRCA1 and 53BP1 to DSB sites[28, 29].

Our conclusion that RNF138 association to DNA damage involved in homologous recombination repair is also supported by resent works showing that RNF138 promote the homologous recombination repair[30, 31].

Formational and regulatory mechanisms of Rad51 filament are still poorly understood. Our findings suggest protein ubiquitylation post-translational modifications are involved in regulating HR DNA damage repair. During HR repair process, DNA damage was sensed and mediated by the MRE11-RAD50-NBS1 complex (MRN) and related proteins [14]. Next, heterotrimetic replication protein A (RPA) is accumulated to the ssDNA ends. Meanwhile, non-degradative ubiquitylation on DNA damage-related proteins are induced, which is regulated by the poly-ubiquitin chains signal on RAD51D. Further ubiquitylation of RAD51D is required to the stable recruitment of the complex of RAD51 paralogs which in turn facilitate the invasion of RAD51 filament in homologous pairs by replacing the PRA (Fig 6G). We speculate these results provide the necessary explanation for the molecular mechanisms of RAD51 filament formation in homologous recombination repair.
